# Cervical Abnormalities Are More Common among Indigenous than Other Australian Women: A Retrospective Record-Linkage Study, 2000–2011

**DOI:** 10.1371/journal.pone.0150473

**Published:** 2016-04-11

**Authors:** Lisa J. Whop, Peter Baade, Gail Garvey, Joan Cunningham, Julia M. L. Brotherton, Kamalini Lokuge, Patricia C. Valery, Dianne L. O’Connell, Karen Canfell, Abbey Diaz, David Roder, Dorota M. Gertig, Suzanne P. Moore, John R. Condon

**Affiliations:** 1 Epidemiology and Health Systems Division, Menzies School of Health Research, Charles Darwin University, Darwin, Brisbane, Australia; 2 Cancer Council Queensland, Brisbane, Australia; 3 Victorian Cytology Service Inc, Melbourne, Victoria, Australia; 4 School of Population and Global Health, University of Melbourne, Melbourne, Victoria, Australia; 5 National Centre of Epidemiology and Public Health, Australian National University, Canberra, Australia; 6 QIMR Berghofer Medical Research Institute, Brisbane, Queensland, Australia; 7 Cancer Research Division, Cancer Council New South Wales, Sydney, New South Wales, Australia; 8 School of Public Health, University of Sydney, Sydney, New South Wales, Australia; 9 School of Medicine and Public Health, University of Newcastle, Newcastle, New South Wales, Australia; 10 Centre for Population Health Research, University of South Australia, Adelaide, South Australia, Australia; State University of Maringá/Universidade Estadual de Maringá, BRAZIL

## Abstract

Indigenous Australian women have much higher incidence of cervical cancer compared to non-Indigenous women. Despite an organised cervical screening program introduced 25 years ago, a paucity of Indigenous-identified data in Pap Smear Registers remains. Prevalence of cervical abnormalities detected among the screened Indigenous population has not previously been reported. We conducted a retrospective cohort study of population-based linked health records for 1,334,795 female Queensland residents aged 20–69 years who had one or more Pap smears during 2000–2011; from linked hospital records 23,483 were identified as Indigenous. Prevalence was calculated separately for Indigenous and non-Indigenous women, for cytology-detected low-grade (cLGA) and high-grade abnormalities (cHGA), and histologically confirmed high-grade abnormalities (hHGA). Odds ratios (OR) were estimated from logistic regression analysis. In 2010–2011 the prevalence of hHGA among Indigenous women (16.6 per 1000 women screened, 95% confidence interval [CI] 14.6–18.9) was twice that of non-Indigenous women (7.5 per 1000 women screened, CI 7.3–7.7). Adjusted for age, area-level disadvantage and place of residence, Indigenous women had higher prevalence of cLGA (OR 1.4, CI 1.3–1.4), cHGA (OR 2.2, CI 2.1–2.3) and hHGA (OR 2.0, CI 1.9–2.1). Our findings show that Indigenous women recorded on the Pap Smear Register have much higher prevalence for cLGA, cHGA and hHGA compared to non-Indigenous women. The renewed cervical screening program, to be implemented in 2017, offers opportunities to reduce the burden of abnormalities and invasive cancer among Indigenous women and address long-standing data deficiencies.

## Introduction

The Australian National Cervical Screening Program (NCSP), introduced in 1991, recommends routine two-year screening by Papanicolau (Pap) smears for women aged 20 to 69 years who have ever been sexually active, regardless of human papillomavirus (HPV) vaccination status, ethnicity, sexual orientation or religion [1]. The major aim of the NCSP is to detect and treat cervical abnormalities prior to progression to invasive cervical cancer. A national system of state-based Pap Smear Registers (PSR) systematically records results of cervical screening, performs a recall and reminder function for women and their primary care providers, and a safety net function for follow up of screen-detected abnormalities. PSRs provide data for national reporting on screening participation, prevalence of abnormalities, outcomes after an abnormal Pap smear and other program quality indicators [2].

Low-grade abnormalities (LGA), indicated by a cytological diagnosis of a low-grade squamous intraepithelial lesion on a Pap smear, are common among young women and usually represent a cellular response to acute HPV infection [3]. Most LGAs resolve without treatment because the infection is cleared; a repeat smear after 6–12 months is currently recommended (unless a woman is aged over 30 years without a recent normal screening history), because persistent infection increases the risk of a high-grade abnormality being present [4–6]. High-grade abnormalities (HGA), as indicated by a cytological detection of a high-grade squamous intraepithelial lesion or a glandular abnormality, can indicate a true precancerous abnormality of the cervix (cervical intraepithelial neoplasia [CIN] grade 3 or adenocarcinoma in situ) or possibly a CIN grade 2 lesion, which is a diagnostic classification that includes both florid low-grade disease and grade 3 disease [7]. Since it is not possible to determine which high-grade lesions will eventually progress to cancer and which will resolve, all are investigated and treatment of confirmed high grade lesions is recommended by the current National Health and Medical Research Council guidelines (p.53) [4,8]. Prompt follow-up of cytology predicted HGAs by colposcopy and biopsy is required to confirm the diagnosis prior to treating the lesion [4].

Aboriginal and Torres Strait Islander women, hereafter respectfully referred to as Indigenous Australian women, have cervical cancer incidence and mortality rates two and four times higher than their non-Indigenous counterparts [9,10]. The NCSP cannot report on screening participation, abnormalities or outcomes for Indigenous women because pathology report forms (the source of information for PSRs) do not include Indigenous status [10–12].

Recently, using record-linkage methods [13], we have reported lower cervical screening participation rates among Indigenous women compared to non-Indigenous women in Queensland [14]. We report for the first time the prevalence of cervical abnormalities for Indigenous women compared to non-Indigenous women participating in cervical screening in Queensland, where 28.5% of the total Australian Indigenous population residing in this Australian state.

## Methods

### Data Sources

The detailed data extraction and linkage methods have been described previously [13]. Briefly, the dataset included linked records from the Queensland PSR and the Queensland Hospital Admitted Patient Data Collections (QHAPDC). The Queensland PSR was used to identify women resident in Queensland aged 20–69 years (at the time of testing) who had a Pap smear between February 1999 (the start of the Queensland PSR) and December 2011, and had not opted to be excluded from the Queensland PSR. Variables obtained from the Queensland PSR included: date of birth (mm/yyyy); place of residence (suburb and postcode); test date; type (cytology or histology); and result. An Indigenous identifier was assigned to the PSR cohort by linking to a QHAPDC extract of women aged 20–69 years who had ever been identified as Indigenous when admitted to a Queensland public hospital during 1995–2011. The QHAPDC has reasonably high accuracy of Indigenous status: in 2011/12, 87% (95% CI 84–91) of Indigenous inpatients were correctly identified as Indigenous in hospital records (compared to self-identification) [15]. Women who linked to at least one QHAPDC record were identified as Indigenous if at least 50% of their QHAPDC records identified them as such [13,16]. Those who did not match to at least one QHAPDC extract record, or had fewer than 50% of their QHAPDC records identified as Indigenous, were assumed to be non-Indigenous. Women were excluded from the study if they had insufficient details to determine the statistical local area (SLA) of residence within Queensland for at least one Pap smear.

### Outcome Measures

Abnormal Pap smear results were categorised according to the Australian Modified Bethesda System 2004 as: low-grade abnormality (LGA: possible or definitive low-grade squamous intraepithelial lesion detected at cytology [includes previous terminology of atypical squamous cells of undetermined significance]); or high-grade abnormality (HGA: prediction of CIN 2 or higher, adenocarcinoma *in situ*, or invasive cancers [includes previous terminology of atypical squamous cells, possible high-grade lesion]), consistent with current national reporting [4]. Cytology-detected LGA and HGA are hereafter referred to as cLGA and cHGA, respectively, to distinguish them from histologically confirmed HGA (hHGA). We categorised women as having an hHGA if there was a record of hHGA within six months after the date of a Pap smear. Women with an HGA histology report who had not had a cytology test within the previous six months were excluded because these tests may have resulted from investigations other than cervical screening.

Location of residence based on suburb and postcode at the time of Pap smear was mapped to the 2011 SLA boundaries. If the address information for a specific Pap smear was insufficient to determine SLA, then information from the closest adjacent record for the same woman was used. SLAs were grouped into five categories from major city to very remote [17]. We assigned an area-based measure of socioeconomic disadvantage to each woman based on the SLA of place of residence using the Index of Relative Socio-Economic Advantage and Disadvantage, with Queensland population-based quintiles from most disadvantaged to most affluent [18].

#### Statistical analyses

Demographic characteristics are presented as medians (with interquartile ranges [IQR]) for non-normally distributed continuous variables and as frequencies and percentages for categorical variables. Proportions for different groups of women were compared using chi-squared tests. Prevalence of hHGA was determined by dividing the number of hHGA in each two-year calendar period (2000–2001 to 2010–2011) by the number of women who were screened in the corresponding two-year period and directly age-standardised based on the 2001 Australian Estimated Resident Population and expressed per 1000 women [19]. Simple linear regression was used to graphically present the association of age-standardised hHGA prevalence and time period stratified by Indigenous status.

Logistic regression was used to quantify the association (as odds ratios) between independent variables of interest and the prevalence of each of cLGA, cHGA and hHGA. The regression models for each outcome included five a priori independent categorical variables of interest (the ‘main effects’ model): Indigenous status; age at time of test, in age-groups of 20–29, 30–39, 40–49 (reference category), 50–59 and 60–69 years; two-year calendar periods from 2000–2001 (reference category) to 2010–2011; place of residence (inner regional as the reference category); and disadvantage quintiles (quintile 3 as the reference category). An interaction between each independent variable and Indigenous status was assessed, but these were not included in the final models because inclusion of the individual interaction terms did not change the estimates for the other a priori variables substantially. To examine if prevalence of abnormalities within each age-group was changing over time, we fitted separate logistic models for each age-group for cLGA and hHGA, including two-year calendar periods as an ordinal variable and Indigenous status, area-level disadvantage and place of residence. We assessed if temporal trends differed by Indigenous status by fitting an interaction term.

To assess whether hHGA was more common for women who had not previously had a Pap smear, we selected women aged 30–69 years who had a Pap smear in 2010–2011 and dichotomised their screening history into ‘yes previous screen’ or ‘no previous screen’ using a ten-year look-back period (2000–2009). Of these women, we determined how many had an hHGA up to six months after their Pap smear in 2010–2011.

Given the non-independence of multiple tests carried out for each woman, we accounted for clustering (i.e. treating each woman as a cluster) in the logistic models and assessed the overall model fit. Because the likelihood ratio test was not appropriate when accounting for clustering, we used the Wald chi-squared test to determine overall significance of each covariate and interaction term. Joint chi-squared tests were used to assess the contribution of each variable to model fit, and the Z test to assess the significance of individual coefficients within the logistic model.

All analyses were conducted using Stata (Version 14.0; Stata Corporation, College Station, TX) [20].

The Human Research Ethics Committees (HREC) of Queensland Health (Far North Queensland HREC HREC/15/QCH/19-957), the Northern Territory Department of Health & Menzies School of Health Research (HOMER-2012-1737) and Charles Darwin University (H12093) approved the study along with the Queensland Research Linkage Group, data custodians, and director general of Queensland Health to access and link records. The research team received a de-identified linked dataset and therefore was unable to obtain individual consent from participants. This process was approved by the aforementioned ethics committees.

## Results

We excluded 1545 women with conflicting dates of birth, 3174 women with missing address details (a total of 11,072 Pap tests) and 518 women with hHGA only. Our final cohort included 1,334,795 women with 4,565,250 Pap smears from 2000–2011. There were 26,829 Indigenous women (2.0%) identified in the PSR cohort with 87,372 Pap smears. The median number of Pap smears per woman was similar for Indigenous and non-Indigenous women (3, IQR 1–5). Similar proportions of Indigenous and non-Indigenous women had only one (26.9% vs 26.6%), two or three (35.4% vs 30.9%) and four or more (37.7% vs 42.4%) Pap smears during the study period. The demographic details of women at their first recorded Pap smear are summarised in Table 1. Indigenous women compared to non-Indigenous women were more likely to be younger (median age 34, IQR 27–43 vs 40, IQR 30–50), live outside major cities and live in less affluent areas.

**Table 1:** Characteristics of Indigenous and non-Indigenous women at their first recorded Pap smear.

	Indigenous		Non-Indigenous		p^a^
Variables	N	%	N	%	
**Totals**	26,829	2.0	1,307,966	98.0	
**Age group (years)**					
20–29	13,104	48.8	476,371	36.4	
30–39	6790	25.3	388,932	25.9	
40–49	4107	15.3	258,661	19.8	
50–59	1982	7.4	158,310	12.1	
60–69	846	3.2	75,692	5.8	<0.001
**Area-level disadvantage**					
Q1 (most disadvantaged)	7358	27.4	148,552	11.4	
Q2	9281	34.6	285,133	21.8	
Q3	4877	18.2	301,630	23.1	
Q4	4090	15.2	326,672	25.0	
Q5 (most affluent)	1223	4.6	245,979	18.8	<0.001
**Place of residence**					
Major city	6700	25.0	812,894	62.2	
Inner regional	4259	15.9	252,875	19.3	
Outer regional	10,593	39.5	218,651	16.7	
Remote	2129	7.9	14,611	1.1	
Very remote	3148	11.7	8935	0.7	<0.001

^a^Chi-squared test of association for categorical variables

The proportions of abnormal tests by socio-demographic variables for Indigenous and non-Indigenous women are shown in Table 2. Greater proportions of tests for Indigenous women compared with non-Indigenous women had cLGA and cHGA (p<0.001) overall and this was consistent by age-group, area-level disadvantage and place of residence.

**Table 2:** Demographic characteristics of Indigenous and non-Indigenous women’s Pap smears by abnormality, 2000–2011.

	Indigenous			Non-Indigenous		
Variables	N	% cLGA	% cHGA	N	% cLGA	% cHGA^a^
**No. Pap smears**	87,372	6.6	3.2	4,477,878	4.3	1.2
**Age group (years)**						
20–24	15,485	13.4	5.7	481,149	10.9	2.3
25–29	15,897	8.5	5.2	554,668	6.8	2.2
30–39	26,854	6.0	3.1	1,193,327	4.3	1.4
40–49	16,659	4.7	2.1	1,080,806	3.7	0.7
50–59	8619	3.4	1.3	765,180	2.8	0.5
60–69	3 858	3.4	1.5	402,748	2.0	0.5
**Area-level disadvantage**						
Q1	24,546	7.2	3.6	501,561	4.5	1.2
Q2	30,114	7.0	3.7	996,147	4.6	1.3
Q3	15,363	7.2	3.3	1,042,366	4.6	1.2
Q4	13,164	7.4	3.6	1,080,243	4.8	1.2
Q5 (most affluent)	4185	6.9	2.7	857,561	4.8	1.1
**Place of residence**						
Major cities	21,334	7.0	3.1	2,745,136	4.8	1.1
Inner regional	13,503	7.1	3.4	895,540	4.2	1.2
Outer regional	35,060	7.0	3.7	761,111	4.9	1.3
Remote	6609	8.4	4.4	46,932	5.0	1.6
Very remote	10,866	7.0	3.3	29,159	4.9	1.7

Notes:

1. Table 2 shows the proportion of abnormal results from the total number of tests by Indigenous status (excluding tests that were taken in the same year with the same result).

2. cLGA: Cytology detected low-grade abnormality; cHGA: cytology detected high-grade abnormality.

^a^ Statistically significant chi-squared test for association, p<0.001 comparing the proportion of abnormal Pap smears among Indigenous women and the proportion of abnormal Pap smears among non-Indigenous women.

The odds of Indigenous women having cLGA (OR 1.7, 95% CI 1.6–1.7), cHGA (OR 2.8, CI 2.7–3.0) and hHGA (OR 2.6, CI 2.5–2.8) were higher than non-Indigenous women after adjusting only for year of Pap smear. The odds remained higher when adjusted further for area-level disadvantage, place of residence and age-group, for example, cLGA OR 1.4, CI 1.3–1.4 (Table 3).

**Table 3:** Association of Indigenous status, calendar period, place of residence, area-level disadvantage and age with risk of cytology detected low-grade abnormalities and cytological and histological high-grade abnormalities including cervical cancer.

	cLGA	cHGA	hHGA
Variables	Adjusted ORs (95% CI)	Adjusted ORs (95% CI)	Adjusted ORs (95% CI)
**Indigenous status**			
Indigenous women	1.38 (1.33–1.43)	2.16 (2.05–2.27)	1.98 (1.87–2.10)
**Year**			
2000–2001	1.00	1.00	1.00
2002–2003	0.99 (0.98–1.01)	1.04 (1.00–1.07)	1.07 (1.03–1.11)
2004–2005	0.99 (0.97–1.00)	1.19 (1.15–1.23)	0.98 (0.94–1.01)
2006–2007	0.82 (0.80–0.83)	1.23 (1.19–1.287)	0.92 (0.88–0.96)
2008–2009	0.65 (0.64–0.66)	1.19 (1.15–1.23)	0.94 (0.91–0.98)
2010–2011	0.52 (0.57–0.59)	1.25 (1.25–1.33)	1.02 (0.98–1.06)
**Area-level disadvantage**			
Q1	1.00 (0.98–1.02)	1.06 (1.02–1.10)	1.04 (1.00–1.09)
Q2	1.01 (0.99–1.03)	1.06 (1.03–1.09)	1.08 (1.04–1.12)
Q3	1.00	1.00	1.00
Q4	1.01 (0.99–1.03)	1.02 (0.99–1.05)	0.97 (0.94–1.00)
Q5 (most affluent)	1.00 (0.98–1.02)	0.90 (0.87–0.93)	0.94 (0.88–1.02)
**Place of residence**			
Major city	1.09 (1.08–1.11)	0.99 (0.96–1.02)	1.02 (0.98–1.05)
Inner regional	1.00	1.00	1.00
Outer regional	1.12 (1.10–1.14)	1.09 (1.05–1.12)	1.07 (1.03–1.11)
Remote	1.13 (1.08–1.18)	1.24 (1.15–1.34)	1.18 (1.08–1.30)
Very remote	1.09 (1.04–1.16)	1.11 (1.02–1.22)	1.04(0.93–1.16)
**Age group (years)**			
20–29	2.53 (2.50–2.57)	3.15 (2.05–2.27)	4.20 (4.05–4.36)
30–39	1.18 (1.16–1.20)	1.87 (1.81–1.92)	2.30 (2.21–2.39)
40–49	1.00	1.00	1.00
50–59	0.75 (0.74–0.77)	0.70 (0.67–0.73)	0.53 (0.50–0.56)
60–69	0.52 (0.50–0.53)	0.59 (0.56–0.63)	0.42 (0.39–0.46)

Notes:

1. Binary logistic regression model comparing odds of having an abnormality adjusted for all a priori variables in the table. Clustering of Pap smears for women was accounted for in each model.

2. Table 3 presents OR, Odd Ratios: 95% C.I., 95% Confidence Intervals

3. Each independent variable in the table had overall p<0.001 and each outcome variable (type of abnormality) p<0.001.

The prevalence of cLGA decreased after 2005; was lower for older than younger women, but was not associated with area-level disadvantage. Compared to women in inner regional areas, women living in all other areas had a higher prevalence of cLGA. For all women, cLGA prevalence significantly decreased over time in all age-groups (Table 4). The prevalence of cHGA increased over time, was lower in the most affluent area, and for older women, but was higher in outer regional, remote and very remote areas (Table 3).

**Table 4:** Time trends in prevalence of cytology low-grade and histologically confirmed high-grade abnormalities by age-group, 2000–2011.

	cLGA	hHGA	
Age-group^a^	All women^b^	Indigenous	Non-Indigenous
20–29	0.94 (0.94–0.94)	1.07 (1.03–1.12)	1.00 (0.99–1.01)
30–39	0.89 (0.88–0.85)	0.99 (0.93–1.05)	0.99 (0.98–1.01)
40–49	0.84 (0.84–0.85)	1.03 (0.93–1.14)	0.96 (0.94–0.98)
50–59	0.81 (0.81–0.82)	0.92 (0.76–1.12)	0.94 (0.91–0.97)
60–69	0.81 (0.80–0.82)	0.84 (0.68–1.02)	0.91 (0.87–0.96)

Notes:

1. Table 4 reports OR (Odd Ratios) for a two-year increase in time; followed by 95% CI (Confidence Intervals).

2. cLGA: Cytology detected low-grade abnormality; cHGA: cytology detected high-grade abnormality.

^a^each model included Indigenous status, area-level disadvantage and place of residence and an interaction term for Indigenous status and year.

^b^ORs were similar for Indigenous and non-Indigenous women for cLGAs over time and therefore have been presented for all women combined.

**Fig 1:**
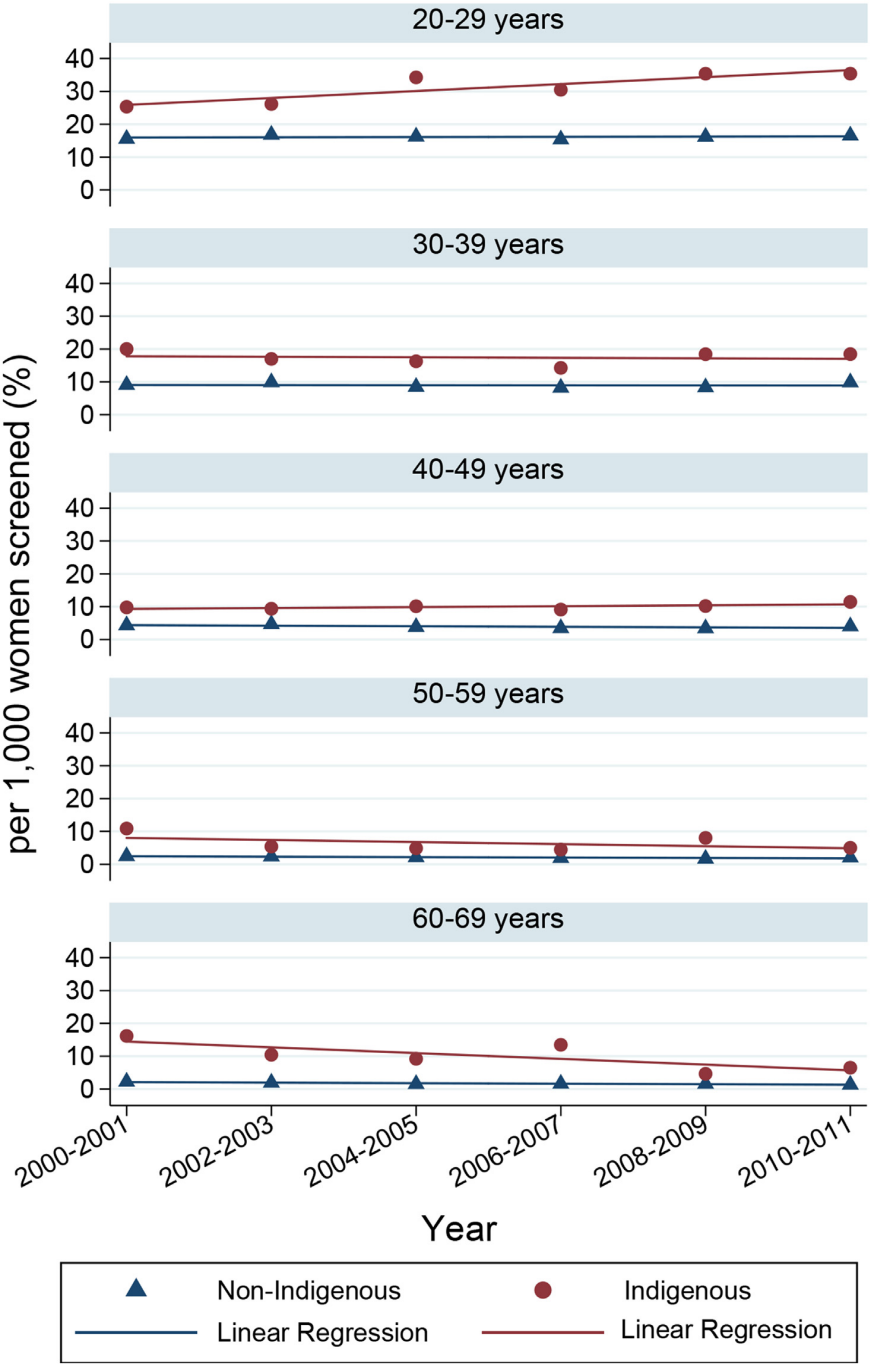
Prevalence of histologically confirmed high-grade abnormalities per 1000 women screened, by age-group and Indigenous status, 2000–2011.

The prevalence of hHGA was higher among Indigenous (16.5 per 1000 women screened, CI 13.9–19.5) than non-Indigenous women (7.3 per 1000 women screened, CI 7.1–7.6), in 2000–2001 and remained relatively constant in both groups up to 2010–2011 (16.6 per 1000 women screened, CI 14.6–18.9 and 7.5 per 1000 women screened, CI 7.3–7.7, respectively). Prevalence was higher for Indigenous women in each age-group (Fig 1). In the 20–29 year age-group, prevalence of hHGA increased over time for Indigenous women only and multivariable analysis confirmed this was a statistically significant trend (Table 4). There were no significant changes in prevalence of hHGA over time for other age-groups among Indigenous women. Among non-Indigenous women, hHGA prevalence decreased significantly in 40–69 year olds.

In 2010–2011, 548,549 women aged 30–69 years had a Pap smear, of whom 1.6% were Indigenous. hHGA was more common among women without a Pap smear history (unadjusted prevalence Indigenous: 36.6 per 1000 women without and 11.3 per 1000 with screening history; non-Indigenous: 10.7 per 1000 without and 4.5 per 1000 with screening history).

We carried out a sensitivity analysis by re-running all models and estimating hHGA prevalence by excluding cervical cancer in the definition of the outcome variable; there were no differences in these and the main results for the prevalence of hHGA (comparison data not shown). We also calculated a histology detection rate, based on the number of HGAs reported by histology, using the method as reported by the Australian Institute of Health and Welfare [21]: the detection rate was 23 and 9 per 1000 women screened in 2011 for Indigenous women and non-Indigenous women, respectively.

## Discussion

Indigenous women had markedly higher prevalence of cytology-detected and histology-confirmed cervical abnormalities than non-Indigenous women. The prevalence of cHGA increased over time for Indigenous women only. Prevalence of cLGA decreased over time for both groups of women.

We were able to identify Indigenous women in the PSR only if women had been a public hospital inpatient between 1995 and 2011 [13]. Women admitted to hospital may differ from those who were not. Indirect evidence suggests that almost all Indigenous women in the age-range for our study would have been admitted to a public hospital over the 17 year period that we used to identify Indigenous women [13], so it is likely that our results apply to almost all screening women. Queensland has the second highest proportion of Indigenous people in its population and they have widely varied geographic and socioeconomic characteristics. Therefore, it is probable that the results for Queensland be applicable to the broader Australian Indigenous population.

The availability of individual-level PSR data facilitated a more precise method for estimating the prevalence of hHGA than reported previously [21]. This previous method calculated hHGA prevalence from aggregated data supplied by PSRs: number of hHGAs (excluding cancer) divided by the number of women screened. We calculated hHGA prevalence directly for screened women (i.e. including only hHGA/cervical cancer if there was a record of a recent Pap smear) using individual-level data. This increased precision in estimating hHGA prevalence in screened women provides greater validity of our study findings. Differences in estimation methods mean comparisons with previously published estimates requires some caution [22].

Participation in cervical screening of Indigenous women is significantly less than that of non-Indigenous women in Queensland, a known risk factor for cervical cancer [14]. Increasing screening among Indigenous women would likely reduce HGAs. We did not have data about other risk factors such as persistent HPV infection, lower individual-level socio-economic status, lower education level, smoking, possible dietary deficiencies, oral contraception, age at first intercourse and early and higher parity [23–27]. The prevalence of HPV types 16 and 18 has previously been reported to be similar for Indigenous and non-Indigenous women and more recently similar age at sexual debut has been reported [28,29]. Some evidence indicates that Indigenous women have higher prevalence of other oncogenic HPV types in the 36–40 year age-group [29]. Indigenous women in this cohort had much higher prevalence of LGAs—a known marker of productive HPV infection—and hHGAs, of which 50–60% are caused by HPV types 16 and 18 [3,30]. This suggests that a difference in HPV infection patterns may exist. Young Indigenous women have higher smoking rates [2], lower age at parity and higher fertility rates [31], less oral contraceptive use [29] and are more likely to live in areas of disadvantage [2]. It is crucial to understand the possible role of these factors in the increased risk of HGAs developing (or not regressing) among Indigenous women.

The Australian Government has announced changes to the current NCSP which will shift to a new program (the ‘Renewal’) in May 2017 using primary HPV testing every five years for women aged 25–74 years [32,33]. Starting screening at age 25 is consistent with international reports showing that screening and treatment of HGAs below the age of 25 is not effective at preventing cancer [34], and will occur in the context of the significant decline in high-grade lesions in 20–24 year olds following the 2007 introduction of the national HPV vaccination program in Australia [10,35]. Given our findings it is timely and important that the Renewal helps address the needs of Indigenous women. High prevalence of HGAs among Indigenous women has previously been reported among Indigenous women in remote areas [22]. Increasing the uptake of HPV vaccination for Indigenous children must be a priority. Although some evidence indicates coverage and/or dose completion rates for Indigenous female adolescents during the catch-up phase were lower than for non-Indigenous female adolescents [36], reassuringly, early vaccination program outcomes in terms of falls in presentations for anogenital warts (caused by HPV types also included in the vaccine) have been similar for 15–25 year old Indigenous and non-Indigenous females [37]. Over the longer term, it is hoped that current school-based routine HPV vaccination, which now includes boys as well as girls, will encourage relatively high ongoing coverage overall and that this mode of delivery will reduce inequities in outcomes for Indigenous women.

The Renewal will provide the option of self-collection of HPV samples for under-screened and never-screened women, which is supported by international evidence that self-collection can increase screening among these groups [38]. Other opportunities to increase screening include an invitation to commence screening at a woman’s 25^th^ birthday using a call-and-recall system inviting women to rescreen, rather than the current reminder-based screening system. Possible mediums of invitations could be considered such as using SMS or email or letter and the inclusion of educational resources and information.

Evidence suggests that there has been some reduction in the incidence of cervical cancer over time among Indigenous women [9,10,39], indicating that efforts to reduce the burden of cervical cancer for Indigenous women have been somewhat effective. For over two decades the NCSP has been unable to report on the impacts of the program for Indigenous Australian women on a wide range of outcomes [11,12,21]. The Renewal program must take the critical opportunity to ensure that Indigenous women are able to be recorded in the proposed national registry. Our study provides benchmark measures giving an opportunity to develop and assess the effectiveness of interventions designed to increase the number of women who are followed up for a serious abnormality and reduce the severity of those outcomes.

Internationally, Australia has been at the forefront of cervical cancer prevention. However, below the surface of this success, Indigenous Australian women carry a disproportionate burden of disease, comparable to many countries without the resources available in Australia. The Renewal of the cervical screening program provides the opportunity to overcome long standing data deficiencies and should consider the Indigenous-specific patterns and trends identified in our study. It is imperative that any major changes to current screening practices prioritise Indigenous women. Failure to do so will result in further disenfranchisement and disadvantage of Indigenous women, their families and their communities.
